# How to Use Therapeutic Drug Monitoring for Comorbidities Management in This Case

**DOI:** 10.1192/j.eurpsy.2025.229

**Published:** 2025-08-26

**Authors:** J. Benninghoff

**Affiliations:** Zentrum für Altersmedizin und Entwicklungsstörungen - ZfAE, kbo, München, Germany

## Abstract

**Abstract:**

Therapeutic Drug Monitoring (TDM) plays a crucial role in optimizing treatment for patients with psychiatric disorders and comorbid medical conditions, particularly in the elderly. This talk will focus on the practical application of TDM in managing polypharmacy and drug-drug interactions in a geriatric patient with mood disorders. Using a case-based approach, the discussion will highlight how TDM can guide dose adjustments, improve treatment efficacy, and minimize adverse effects in patients with multiple comorbidities.

Emphasizing real-world clinical challenges, this session will provide practical insights into integrating TDM into psychiatric practice, ensuring safer and more effective pharmacotherapy. Audience interaction will be encouraged through live voting and discussion to enhance learning and application in daily clinical settings.

**Disclosure of Interest:**

None Declared

